# Author Correction: National analysis of cancer mortality and proximity to nuclear power plants in the United States

**DOI:** 10.1038/s41467-026-72052-0

**Published:** 2026-04-13

**Authors:** Yazan Alwadi, Barrak Alahmad, Carolina L. Zilli Vieira, Philip J. Landrigan, David C. Christiani, Eric Garshick, Marco Kaltofen, Brent Coull, Joel Schwartz, John S. Evans, Petros Koutrakis

**Affiliations:** 1 Environmental Health Department, Harvard T.H. Chan School of Public Health, Boston, MA USA; 2 Boston College, Chestnut Hill, MA USA; 3 Centre Scientifique de Monaco, Monaco, Monaco; 4 Division of Pulmonary and Critical Care Medicine, Department of Medicine, Massachusetts General Hospital, Boston, MA USA; 5 Harvard Medical School, Boston, MA USA; 6 Pulmonary, Allergy, Sleep, and Critical Care Medicine Section, Medical Service, VA Boston Healthcare System, Boston, MA USA; 7 Boston Chemical Data Corp, Natick, MA USA; 8 Department of Biostatistics, Harvard T.H. Chan School of Public Health, Boston, MA USA

**Keywords:** Environmental impact, Risk factors

Correction to: *Nature Communications* 10.1038/s41467-026-69285-4, published online 23 February 2026

In the version of this article initially published, there was a typographical error in the title to Fig. 5, where the time span now reading “1980–2018” appeared originally as “2000–2018.” The figure title is amended in the HTML and PDF versions of the article.

