# Supplementary material for: National analysis of cancer mortality and proximity to nuclear power plants in the United States
Source: Nat Commun. 2026 Feb 23;17:1560. doi: 10.1038/s41467-026-69285-4 (PMC12929679; doi:10.1038/s41467-026-69285-4)
Supplement: Supplementary file 1 — Supplementary Information [file 41467_2026_69285_MOESM1_ESM.pdf]

Table S1. Total cancer deaths in all counties within 200 km of an operational power plant for the duration of 2000 to 2018.

SEX	AGE GROUP	TOTAL CANCER DEATHS
**FEMALE**	35_44	97,536
**FEMALE**	45_54	311,442
**FEMALE**	55_64	624,238
**FEMALE**	65_74	878,866
**FEMALE**	75_84	1,010,316
**FEMALE**	85+	670,248
**MALE**	35_44	73,665
**MALE**	45_54	307,074
**MALE**	55_64	746,782
**MALE**	65_74	1,062,167
**MALE**	75_84	1,105,497
**MALE**	85+	559,185

Table S2. Annual covariates used in the statistical models (2000-2018).

Variable	Data source
**Education below high school, %**	United States Census American Community Survey
**Median household income, $**	United States Census American Community Survey
**Below federal poverty level, %**	United States Census American Community Survey
**White, %**	United States Census American Community Survey
**Population density, ppl/km^2**	NASA Socioeconomic Data and Applications Center
**Average temperature, degrees ^{o}C**	National Oceanic and Atmospheric Administration
**Average relative humidity, %**	National Oceanic and Atmospheric Administration
**Current smoke, (1-100)**	BRFSS Area Health Resource Files
**Nearest hospital, Km**	ESRI's hospital distribution files
**Age over 65, %**	United States Census American Community Survey
**Ambulance visit, %**	Dartmouth Health Atlas
**Renting, %**	United States Census American Community Survey
**Poverty, %**	United States Census American Community Survey
**Asian, %**	United States Census American Community Survey
**African American, %**	United States Census American Community Survey
**Average BMI**	BRFSS Area Health Resource Files
