# Supplementary material for: National analysis of cancer mortality and proximity to nuclear power plants in the United States
Source: Nat Commun. 2026 Feb 23;17:1560. doi: 10.1038/s41467-026-69285-4 (PMC12929679; doi:10.1038/s41467-026-69285-4)
Supplement: Supplementary file 2 — Description of Additional Supplementary Files [file 41467_2026_69285_MOESM2_ESM.pdf]

Description of Additional Supplementary Information

Supplementary Dataset 1. This dataset contains all power plants situated within 200 km of each county center and operational for at least one year during the period 2000–2018. Variables include plant name, unique identifier, geographic coordinates, plant type, operational status by year, and computed distance from the plant to the county center. This dataset was used to identify counties with potential exposure to emissions or activities associated with nearby power plants.
