# Supplementary material for: National analysis of cancer mortality and proximity to nuclear power plants in the United States
Source: Nat Commun. 2026 Feb 23;17:1560. doi: 10.1038/s41467-026-69285-4 (PMC12929679; doi:10.1038/s41467-026-69285-4)
Supplement: Supplementary file 4 — Reporting Summary [file 41467_2026_69285_MOESM4_ESM.pdf]

nature portfolio

Corresponding author(s): Yazan Alwadi

Last updated by author(s): Oct 29, 2025

Reporting Summary

Nature Portfolio wishes to improve the reproducibility of the work that we publish. This form provides structure for consistency and transparency in reporting. For further information on Nature Portfolio policies, see our Editorial Policies and the Editorial Policy Checklist.

Statistics

For all statistical analyses, confirm that the following items are present in the figure legend, table legend, main text, or Methods section.

n/a	Confirmed	
☐	☒	The exact sample size (n) for each experimental group/condition, given as a discrete number and unit of measurement
☐	☒	A statement on whether measurements were taken from distinct samples or whether the same sample was measured repeatedly
☒	☐	The statistical test(s) used AND whether they are one- or two-sided<br>*Only common tests should be described solely by name; describe more complex techniques in the Methods section.*
☐	☒	A description of all covariates tested
☒	☐	A description of any assumptions or corrections, such as tests of normality and adjustment for multiple comparisons
☐	☒	A full description of the statistical parameters including central tendency (e.g. means) or other basic estimates (e.g. regression coefficient) AND variation (e.g. standard deviation) or associated estimates of uncertainty (e.g. confidence intervals)
☒	☐	For null hypothesis testing, the test statistic (e.g. F, t, r) with confidence intervals, effect sizes, degrees of freedom and P value noted<br>*Give P values as exact values whenever suitable.*
☒	☐	For Bayesian analysis, information on the choice of priors and Markov chain Monte Carlo settings
☒	☐	For hierarchical and complex designs, identification of the appropriate level for tests and full reporting of outcomes
☒	☐	Estimates of effect sizes (e.g. Cohen's d, Pearson's r), indicating how they were calculated

Our web collection on statistics for biologists contains articles on many of the points above.

Software and code

Policy information about availability of computer code

Data collection	No code was used in data collection
Data analysis	All analyses were performed using RStudio (version 2023.09.0+463) with R (version 4.3.2). Statistical modeling, data processing, and visualization were conducted using established functions from the following R packages: geepack for generalized estimating equation (GEE) models, data.table and dplyr for data manipulation, sf and raster for spatial operations, and ggplot2 for figure generation. The workflow integrated these package functions to perform all the calculations.

For manuscripts utilizing custom algorithms or software that are central to the research but not yet described in published literature, software must be made available to editors and reviewers. We strongly encourage code deposition in a community repository (e.g. GitHub). See the Nature Portfolio guidelines for submitting code & software for further information.

Data

Policy information about availability of data

All manuscripts must include a data availability statement. This statement should provide the following information, where applicable:

- Accession codes, unique identifiers, or web links for publicly available datasets
- A description of any restrictions on data availability
- For clinical datasets or third party data, please ensure that the statement adheres to our policy

The cancer mortality data used in this study were obtained from the Centers for Disease Control and Prevention (CDC) and are confidential; therefore, they cannot be publicly shared. Researchers may request access directly from the CDC. All other data sources used in this analysis are publicly available. The aggregated data

underlying all main figures and tables are provided as a Source Data file. Source data are provided with this paper.

Research involving human participants, their data, or biological material

Policy information about studies with human participants or human data. See also policy information about sex, gender (identity/presentation), and sexual orientation and race, ethnicity and racism.

Reporting on sex and gender	Sex-disaggregated analyses were performed using county-level cancer mortality data stratified by biological sex (male and female) as provided by the Centers for Disease Control and Prevention (CDC). The dataset contains no individual-level or gender identity information. Analyses were based solely on biologically defined sex categories reported in official mortality records.
Reporting on race, ethnicity, or other socially relevant groupings	County-level percentages of racial and ethnic composition (White, African American, and Asian) were included as covariates to control for potential confounding. These data were obtained from the U.S. Census American Community Survey and represent self-identified categories in administrative records. Race and ethnicity were not used as proxies for other variables (e.g., socioeconomic status), and all related covariates were analyzed at the aggregated county level.
Population characteristics	The study analyzed aggregated annual cancer mortality data for adults aged 35 years and older, stratified by six age groups (35–44, 45–54, 55–64, 65–74, 75–84, and 85+) and by sex. No individual-level data were used. All analyses were conducted at the county level across the contiguous United States.
Recruitment	No direct recruitment of participants occurred. The analysis used publicly available or restricted-use administrative datasets collected by federal agencies (CDC, U.S. Census, NOAA, and EIA).
Ethics oversight	The study protocol was reviewed by the Institutional Review Board of the Harvard T.H. Chan School of Public Health, which determined that the project does not involve human subjects research as defined by U.S. Department of Health and Human Services and U.S. Food and Drug Administration regulations.

Note that full information on the approval of the study protocol must also be provided in the manuscript.

Field-specific reporting

Please select the one below that is the best fit for your research. If you are not sure, read the appropriate sections before making your selection.

☐ Life sciences ☐ Behavioural & social sciences ☒ Ecological, evolutionary & environmental sciences

For a reference copy of the document with all sections, see nature.com/documents/nr-reporting-summary-flat.pdf

Ecological, evolutionary & environmental sciences study design

All studies must disclose on these points even when the disclosure is negative.

Study description	This ecological study assessed the association between proximity to operational nuclear power plants and all-cancer mortality across U.S. counties from 2000 to 2018. The exposure variable was an annual proximity metric defined as the sum of the inverse distances from all operational nuclear plants within 200 km of each county centroid. Analyses were stratified by sex and six age groups and conducted using generalized estimating equation (GEE) Poisson regression models with county-level covariates.
Research sample	The research sample consisted of all counties within 200 km of at least one operational nuclear power plant in the contiguous United States between 2000 and 2018. The unit of analysis was the county-year-age-sex combination. The study population included adults aged 35 years and older, with annual counts of cancer mortality derived from the U.S. Centers for Disease Control and Prevention (CDC) restricted-use mortality files.
Sampling strategy	No sampling was performed, as the study included all eligible counties within 200 km of any operational nuclear power plant during the study period. The approach ensured full national coverage of the target population without sample-size estimation, providing adequate statistical power for all analyses.
Data collection	All data were obtained from established national administrative and publicly available sources. Cancer mortality data were obtained from the CDC; nuclear plant operational data from the U.S. Energy Information Administration (EIA); meteorological data (temperature and relative humidity) from the National Oceanic and Atmospheric Administration (NOAA); and socioeconomic and demographic covariates from the U.S. Census American Community Survey. No new data were collected by the authors.
Timing and spatial scale	Data covered the years 2000 through 2018, representing 19 annual observations per county. Analyses were conducted at the county level across the contiguous United States, encompassing all counties within 200 km of at least one operational nuclear power plant.
Data exclusions	No Data Exclusions
Reproducibility	The analysis relied on reproducible national datasets from federal agencies. Replication was assessed through extensive sensitivity analyses, including varying proximity thresholds (100–200 km) and proximity averaging windows (2–20 years), which yielded consistent results and confirmed the robustness of the findings.
Randomization	Randomization was not applicable. This was an observational ecological study using administrative data aggregated at the county level, without experimental assignment.

Blinding	Blinding was not applicable. The study used de-identified administrative datasets, and analyses were based entirely on objective, pre-existing records with no investigator involvement in data collection.

Did the study involve field work? ☐ Yes ☒ No

Reporting for specific materials, systems and methods

We require information from authors about some types of materials, experimental systems and methods used in many studies. Here, indicate whether each material, system or method listed is relevant to your study. If you are not sure if a list item applies to your research, read the appropriate section before selecting a response.

Materials & experimental systems

n/a	Involved in the study	
☒	☐	Antibodies
☒	☐	Eukaryotic cell lines
☒	☐	Palaeontology and archaeology
☒	☐	Animals and other organisms
☒	☐	Clinical data
☒	☐	Dual use research of concern
☒	☐	Plants

Methods

n/a	Involved in the study	
☒	☐	ChIP-seq
☒	☐	Flow cytometry
☒	☐	MRI-based neuroimaging

Plants

Seed stocks	Not applicable. No plant materials or biological samples were used in this study.
Novel plant genotypes	Not applicable. The study did not involve any genetic, molecular, or laboratory-based plant work.
Authentication	Not applicable. No plant genotypes or biological specimens were generated or authenticated.
